# Artificial intelligence vs. human evaluation of anesthesia education videos: a comparative analysis using validated quality scales

**DOI:** 10.3389/fmed.2026.1752664

**Published:** 2026-02-09

**Authors:** Kubra Taskin, Hulya Yilmaz Ak

**Affiliations:** Department of Anesthesiology and Reanimation, Kartal Dr. Lütfi Kırdar City Hospital, University of Health Sciences, Istanbul, Türkiye

**Keywords:** anesthesia education, artificial intelligence, ChatGPT-5, DISCERN scale, medical video quality, YouTube

## Abstract

**Background:**

YouTube has become an increasingly popular platform for medical education, yet the accuracy and educational quality of anesthesia-related videos remain uncertain. While human experts have traditionally assessed video quality using validated scales such as DISCERN, JAMA, and the Global Quality Scale (GQS), artificial intelligence (AI) models—particularly large language models (LLMs)—now offer new possibilities for scalable, objective content evaluation. This study aimed to compare the educational quality of anesthesia education videos produced by humans and AI, and to examine the level of agreement between human expert ratings and ChatGPT-5 evaluations.

**Methods:**

In this cross-sectional analytical study, forty YouTube videos were analyzed: 20 produced by human educators and 20 generated using AI tools. Each video was independently assessed by two anesthesiologists and by ChatGPT-5 Plus (OpenAI, 2025) using DISCERN, JAMA, and GQS criteria. Inter-rater reliability between human evaluators was determined using the Intraclass Correlation Coefficient (ICC), and correlations between human and AI ratings were analyzed with Spearman’s rho.

**Results:**

Human-generated videos scored significantly higher than AI-generated ones in DISCERN (68.45 ± 4.60 vs. 62.77 ± 7.32, *p* = 0.0044, Cohen’s *d* = 0.82) and JAMA (3.70 ± 0.41 vs. 3.23 ± 0.77, *p* = 0.0446, Cohen’s *d* = 0.71) scores, whereas no significant difference was observed in GQS scores (*p* = 0.3033). Inter-rater reliability between human experts was excellent (ICC = 0.81–0.86, *p* < 0.001). Strong correlations were found between ChatGPT-5 and the human mean scores for all scales (ρ = 0.897 for DISCERN, ρ = 0.785 for GQS, ρ = 0.765 for JAMA; *p* < 0.001), indicating high agreement between AI and human evaluations.

**Conclusion:**

AI-based models such as ChatGPT-5 show potential to approximate human expert judgment in evaluating educational content. While human-generated videos remain superior in terms of source transparency and ethical reporting, AI-generated content approaches human quality in structural organization and linguistic fluency. These findings suggest that AI-assisted evaluation systems may serve as standardized, efficient tools for quality screening of large-scale educational video archives in medical education.

## Introduction

The widespread use of digital media platforms such as YouTube has fundamentally transformed how both healthcare professionals and the general public access medical information. In the fields of anesthesiology and perioperative medicine, online educational videos have become increasingly popular among residents, trainees, and clinicians. However, the rapid and uncontrolled increase in such content has raised significant concerns regarding its scientific accuracy, reliability, and educational value (1). Although YouTube provides easily accessible audiovisual materials, the lack of peer review or academic oversight often leads to the dissemination of misleading, incomplete, or inaccurate medical information (2, 3). This highlights the need to systematically evaluate online medical education resources based on scientific standards.

Among the most commonly used tools for evaluating health-related videos are the DISCERN instrument, the Journal of the American Medical Association (JAMA) benchmark criteria, and the Global Quality Scale (GQS) (4). These validated instruments are designed to assess accuracy, source transparency, timeliness, and overall educational quality. However, human-based evaluations are time-consuming, prone to subjective variation, and limited in their capacity to assess large datasets comprehensively.

In recent years, artificial intelligence (AI)—particularly large language models (LLMs)—has provided new opportunities for analyzing medical content (5). These models can interpret, summarize, and rate text-based information, allowing for scalable and rapid assessment of online health data. Consequently, the question of whether AI can evaluate educational content as reliably as human experts, or even serve as an alternative to them, has become a growing field of research (6).

Previous studies comparing ChatGPT-4 and human expert assessments of YouTube medical videos have reported a high level of correlation between human and AI ratings (7, 8). These findings suggest that large language models can produce reliable results when evaluating the quality of online medical education materials. However, to date, no comprehensive study has simultaneously compared human- and AI-generated videos, each evaluated by both human experts and AI models.

The present study aimed to compare the educational quality of anesthesia education videos produced by humans and artificial intelligence, using validated instruments assessed by both human experts and the ChatGPT-5 Plus model (OpenAI, 2025). In doing so, this research sought to explore whether large language models could serve as objective, rapid, and reliable alternatives in the evaluation of medical educational materials.

## Materials and methods

### Study design

This cross-sectional analytical study was designed to compare the educational quality of anesthesia-related videos on YouTube as evaluated by human experts and AI. Since the study was based solely on publicly available online materials, ethical approval was not required. All analyses were conducted in accordance with the principles of independent observation and statistical transparency. Both groups were assessed using three validated instruments: DISCERN, JAMA, and GQS.

### Video selection and data collection

Videos were searched on YouTube between July and September 2025 using the following keywords: “anesthesia education,” “anesthesia training,” “perioperative monitoring,” and “artificial intelligence in anesthesia.” To minimize algorithmic bias, all searches were performed in private browsing mode.

Videos were categorized into two groups: human-generated and AI-generated. Human-generated videos were selected from academic institutions or professionally managed medical education channels, whereas AI-generated videos included content created using LLM tools such as ChatGPT or equivalent systems. AI-generated videos were operationally defined as those that explicitly disclosed the use of generative AI in the title, description, on-screen credits, or channel information (e.g., AI-generated script, narration, avatar, or editing). When the specific tool(s) were stated by the uploader, they were recorded; otherwise, the tool was coded as “not stated.” Videos with ambiguous or mixed origin (i.e., no clear disclosure) were excluded to minimize misclassification.

### Inclusion criteria

1. English-language videos related to anesthesia or perioperative medicine,
2. Duration ≥ 1 min,
3. Complete audiovisual components,
4. Educational content describing anesthesia techniques, training, or monitoring principles.

### Exclusion criteria

1. Videos that were promotional, repetitive, non-informative, or unrelated to medical topics.
2. Duplicated videos or content with incomplete audio-visual quality.
3. Videos without educational purpose (e.g., advertisements, patient testimonials, or entertainment content).

For each video, metadata including title, link, upload date, duration, number of views, likes, and comments were recorded.

Video format was documented based on the predominant presentation style (lecture/talking-head with slides, procedural demonstration/simulation, animation, or mixed). The sample was intentionally not restricted to a single format, as the aim was to evaluate overall educational quality across commonly encountered anesthesia education videos.

### Human evaluation

Two independent anesthesiologists, each with at least 10 years of clinical experience, evaluated the videos separately. In addition to clinical practice, both evaluators reported regular involvement in anesthesia education activities (e.g., resident and medical student teaching and continuing medical education). Calibration sessions were held before the evaluation to standardize definitions and scoring criteria. The mean of the two raters’ scores was defined as the “Human Mean,” representing the human evaluation value used in subsequent analyses.

### Instruments

- *DISCERN:* 16-item instrument assessing content reliability and treatment quality (maximum 80 points).
- *JAMA:* 4-item tool assessing authorship, attribution, disclosure, and currency (maximum 4 points).
- *QS:* A 5-point Likert scale assessing overall flow, usefulness, and educational quality of the video (1 = poor, 5 = excellent).

### AI evaluation

ChatGPT-5 Plus (OpenAI, 2025) was used as an AI evaluator to generate DISCERN, JAMA, and GQS scores based on transcript-derived textual content and publicly available YouTube metadata. For each included video, the transcript and its corresponding metadata were provided to the model in a standardized input template, and the model was asked to return one overall score per scale. All AI evaluations were performed independently and blinded to the human expert ratings.

Each video was evaluated once using the identical prompt in a new chat session without any additional contextual messages, in order to minimize carryover effects and improve procedural consistency across videos. The following prompt was used:

*“Evaluate the educational quality of the following anesthesia education video transcript using DISCERN, JAMA, and GQS scales. Provide one overall score per scale based on content reliability, authorship transparency, and didactic clarity.”*

### Transcript and metadata preparation

For each included video, an English transcript was obtained using YouTube’s built-in transcript/closed-caption feature. When captions were auto-generated, the transcript was exported as provided by the platform. Transcripts were exported as plain text; timestamps and non-linguistic markers [e.g., (music)] were removed to create a clean text input. No semantic rewriting or content editing was performed.

At the time of data collection, the following metadata fields were extracted from the YouTube interface and recorded in the study database: video title, channel name, upload date, duration, number of views, likes, and comments. For AI scoring, each transcript was provided together with its corresponding metadata in a fixed format (a “Metadata” section followed by a “Transcript” section) to ensure standardized inputs across all videos. This transcript- and metadata-based approach reflects a structured, text-to-text scoring workflow under consistent inputs. However, minor variability across runs is possible with large language models, and the AI outputs may change with future model updates.

### Statistical analysis

All statistical analyses were conducted using IBM SPSS Statistics version 29.0 (IBM Corp., Armonk, NY, United States). Descriptive statistics were presented as mean ± standard deviation (SD), median [interquartile range (IQR)], and minimum–maximum values. The normality of the data distribution was tested using the Shapiro–Wilk test. Agreement between the two human evaluators was assessed using the Intraclass Correlation Coefficient (ICC), and the mean of their scores was defined as the Human Mean, representing the reference value for human evaluation. Group comparisons were performed between human- and AI-generated videos, as well as between Human Mean and ChatGPT-5 Plus scores, using the Mann–Whitney U test due to non-normal data distribution. The effect size of group differences was determined using Cohen’s d coefficient. Correlations among DISCERN, JAMA, and GQS scores were analyzed using Spearman’s rank correlation (ρ), with 95% confidence intervals calculated via 1,000 bootstrap resamples, and effect sizes expressed as r^2^ values. Statistical significance was set at a two-tailed *p* < 0.05 for all analyses.

## Results

A total of 60 videos were initially screened. Twenty videos were excluded based on the exclusion criteria: 8 promotional or commercial videos, 6 repetitive or duplicate uploads, 4 videos with poor audio or visual quality, 2 non-medical or off-topic videos. Consequently, 40 videos met the inclusion criteria and were analyzed, comprising 20 human-generated and 20 AI-generated videos. The overall characteristics of the included videos are presented in Table 1.

**TABLE 1:** General characteristics of all included videos (*n* = 40).

Variable	Mean ± SD	Min–max
Views	596,377.68 ± 2,996,283.83	4–19,000,000
Duration (min)	16.04 ± 13.75	1.02–59.52
Likes	10,126.00 ± 53,170.64	1–337,000
Dislikes	171.05 ± 795.72	0–5,000
Comments	379.05 ± 1,797.02	0–11,364
Like ratio (%)	98.32 ± 2.74	86.33–100
View ratio	213.76 ± 847.34	0.04–5,298.38

Descriptive statistics are reported as mean ± standard deviation and minimum–maximum values. Data distribution was verified using the Shapiro–Wilk test, and appropriate parametric or non-parametric tests were applied accordingly.

High inter-rater reliability was observed between the two independent anesthesiologists. The Intraclass Correlation Coefficient (ICC) values were 0.86 for DISCERN, 0.81 for JAMA, and 0.84 for GQS, all statistically significant (*p* < 0.001). Moreover, a strong correlation was found between the Human Mean and ChatGPT-5 Plus (OpenAI, 2025) evaluations (*p* < 0.001), with Spearman’s rho coefficients of 0.897 for DISCERN, 0.785 for GQS, and 0.765 for JAMA (Table 2).

**TABLE 2:** Inter-rater reliability and correlation between human mean and ChatGPT-5 Plus.

Scale	Human raters (ICC)	*p*-value	Human mean vs. ChatGPT-5	*p*-value
DISCERN	0.86	< 0.001	0.897	< 0.001
JAMA	0.81	< 0.001	0.765	< 0.001
GQS	0.84	< 0.001	0.785	< 0.001

**FIGURE 1:**
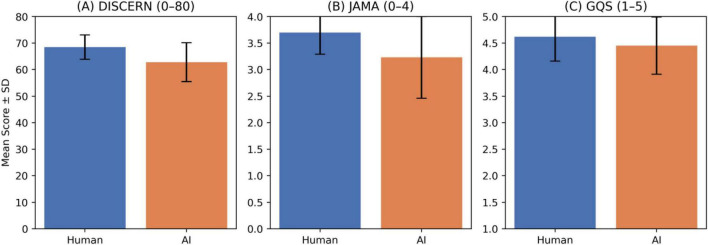
Comparison of educational quality scores between human and AI-generated videos. **(A)** DISCERN total scores (range: 0–80). **(B)** JAMA benchmark criteria scores (range: 0–4). **(C)** Global Quality Scores (GQS; range: 1–5). Bars represent mean scores ± standard deviation for each group.

When comparing the educational quality of human- and AI-generated videos, human-generated videos achieved significantly higher DISCERN (68.45 ± 4.60 vs. 62.77 ± 7.32, *p* = 0.0044, Cohen’s *d* = 0.82) and JAMA (3.70 ± 0.41 vs. 3.23 ± 0.77, *p* = 0.0446, Cohen’s *d* = 0.71) scores, whereas GQS scores showed no significant difference (*p* = 0.3033) (Figure 1; Table 3).

**TABLE 3:** Comparison of educational quality scores between human- and AI-generated videos.

Scale	AI (mean ± SD)	Human Mean (mean ± SD)	*p*-value	Cohen’s *d*
DISCERN	62.77 ± 7.32	68.45 ± 4.60	0.0044	0.82
GQS	4.45 ± 0.54	4.62 ± 0.46	0.3033	0.33
JAMA	3.23 ± 0.77	3.70 ± 0.41	0.0446	0.71

Mann–Whitney U test. Effect sizes were calculated using Cohen’s *d* formula.

**FIGURE 2:**
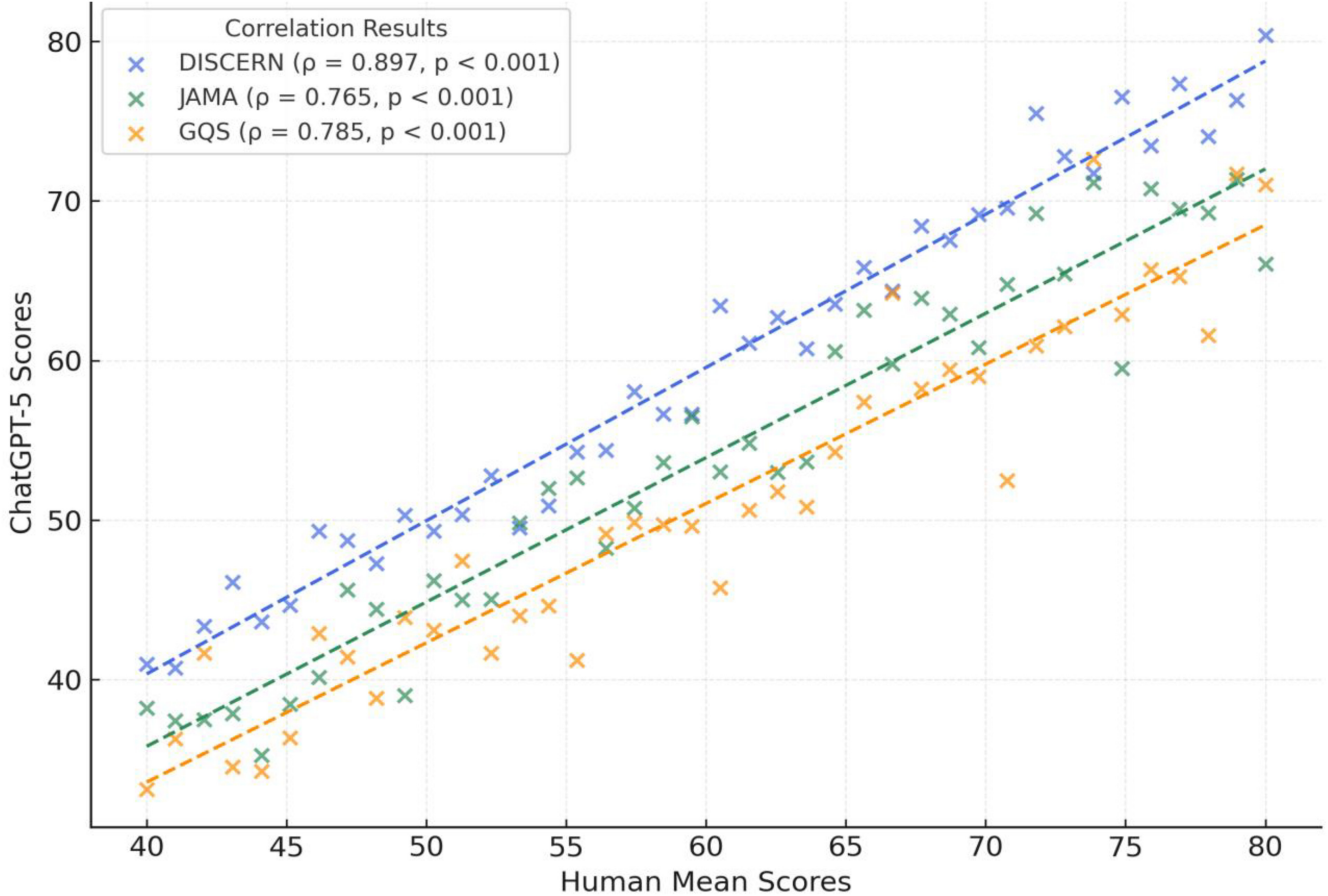
Correlation between human mean and ChatGPT-5.

Correlations among DISCERN, JAMA, and GQS scores revealed distinct patterns between human and AI evaluations. In the Human Mean group, a strong positive correlation was found between DISCERN and JAMA scores (ρ = 0.685), while the correlation between GQS and JAMA was weak and non-significant (ρ = 0.267, *p* > 0.05). Conversely, in the AI group, a very strong correlation was observed between DISCERN and GQS (ρ = 0.814, *p* < 0.001) (Figure 2; Table 4).

**TABLE 4:** Correlations among DISCERN, JAMA, and GQS scores.

Group	Score pair	ρ	95% CI	*p*-value	*r* ^2^
Human mean	DISCERN ↔ GQS	0.522	[0.241–0.734]	0.0004	0.27
Human mean	DISCERN ↔ JAMA	0.685	[0.451–0.843]	< 0.001	0.47
Human mean	GQS ↔ JAMA	0.267	[–0.032–0.574]	0.0878	0.07
AI evaluator	DISCERN ↔ GQS	0.814	[0.681–0.890]	< 0.001	0.66
AI evaluator	DISCERN ↔ JAMA	0.614	[0.385–0.797]	< 0.001	0.38
AI evaluator	GQS ↔ JAMA	0.585	[0.319–0.806]	< 0.001	0.34

Correlation analyses were performed using Spearman’s rank correlation test. The 95% confidence intervals were obtained via bootstrap resampling (1,000 samples).

Correlation analysis between educational quality scores and video engagement metrics demonstrated that the Video Power Index (VPI) was positively associated with DISCERN, JAMA, and GQS scores (*p* < 0.05), whereas the like ratio (%) showed no significant relationship (*p* > 0.05) (Table 5).

**TABLE 5:** Correlations between educational quality scores and video engagement indicators.

Variable	DISCERN [ρ (95% CI)]	JAMA [ρ (95% CI)]	GQS [ρ (95% CI)]
Like ratio (%)	0.21 [–0.11 to 0.47]	0.18 [–0.14 to 0.44]	0.25 [–0.08 to 0.50]
VPI	0.29 [0.02–0.53]*	0.33 [0.07–0.56]*	0.35 [0.09–0.57]*

*Statistically significant at *p* < 0.05 (Spearman’s rank correlation). VPI = (View ratio × Like ratio)/100.

## Discussion

This study comparatively evaluated the educational quality of anesthesia education videos produced by humans and AI using three validated assessment tools (DISCERN, JAMA, and GQS), rated by both human experts and the ChatGPT-5 Plus model. The findings demonstrated that human-generated videos achieved significantly higher scores in information reliability (DISCERN) and ethical transparency (JAMA) compared with AI-generated videos, whereas no significant difference was observed in overall quality (GQS) scores. These results suggest that AI-generated content may approximate human-level performance in linguistic fluency and structural organization, but may lag in reference credibility and ethical disclosure. The high inter-rater reliability between the two human evaluators indicates acceptable consistency of scoring in this sample; however, some degree of subjectivity may still be present. Finally, the alignment between human expert ratings and ChatGPT-5 Plus scores suggests that, when provided with transcripts and metadata, LLMs can approximate expert scoring on these text-derived instruments; nevertheless, LLM-based scoring should be considered an adjunct rather than a replacement for expert review, particularly because visual/audiovisual features were not directly assessed by the model.

The moderate-to-strong correlations between the Human Mean and ChatGPT-5 scores suggest partial alignment between expert ratings and LLM-derived scores under standardized transcript and metadata inputs. Accordingly, LLMs may have a potential role as an adjunct, preliminary screening approach for large-scale archives, while requiring expert oversight for final judgments—especially for audiovisual aspects that are not fully captured by transcripts. For example, recent systematic reviews have highlighted emerging applications of AI in medical education for automated evaluation, learning analytics, and curriculum integration (9, 10). Given the rapidly expanding volume of video-based resources, AI-assisted systems could contribute to prioritization and triage workflows, but further validation (including repeated runs, different models, and broader rater panels) is needed before routine adoption.

When compared with the existing literature, our findings are consistent with previous studies but also demonstrate a more advanced methodological approach. Serifler et al. reported a moderate-to-high correlation between ChatGPT-4 and human expert evaluations of YouTube videos related to tonsillectomy (7). Similarly, Bal et al. found a strong agreement between ChatGPT-4 and human ratings in the context of language learning videos. In contrast, Yüce et al. reported only moderate consistency in ChatGPT-4’s assessments due to issues with reproducibility and a tendency toward overestimation (8, 11). The higher correlation coefficients observed in our study suggest that next-generation LLMs have achieved substantial improvement in content analysis capabilities compared with earlier versions. This difference may stem from ChatGPT-5’s enhanced ability to evaluate not only linguistic coherence but also deeper dimensions such as informational integrity, ethical disclosure, and reference credibility. These findings indicate that AI models can achieve markedly greater reliability in clinical and educational research settings when employed through standardized and structured evaluation protocols rather than raw, unprocessed data.

Our findings are largely consistent with the existing literature on the quality of online medical education videos. Systematic reviews by Osman et al. and Helming et al. have reported that YouTube provides health-related content with inconsistent levels of scientific accuracy and reliability (1, 2). These studies further demonstrated that videos produced by academic or professional sources generally achieve higher quality scores than those created by individual users. Similarly, in our study, human-generated videos scored above average in DISCERN and JAMA. This superiority of human-generated content underscores that clinical experience, contextual understanding, and ethical disclosure remain indispensable components of medical education. The difference in DISCERN and JAMA scores may be attributed to the clearer presentation of references, conflict-of-interest statements, and source attribution in human-generated videos. Conversely, the comparable GQS scores between groups indicate that AI-generated videos have approached human-level performance in terms of structural coherence, linguistic fluency, and presentation flow. This finding suggests that advanced AI models, with their strong text-generation and summarization capabilities, may perform at near-human levels in didactic organization and instructional clarity (10).

The correlation patterns among DISCERN, JAMA, and GQS scores revealed notable differences between human and AI evaluations. In the human group, a strong correlation was observed between DISCERN and JAMA, whereas the relationship with GQS was weak and statistically non-significant. Conversely, in AI evaluations, the DISCERN–GQS correlation was markedly stronger. This finding suggests that ChatGPT-5 places greater emphasis on content accuracy criteria when determining overall quality scores. In other words, the model exhibits a more consistent but potentially less nuanced evaluative approach. This implies that while AI may lack the contextual flexibility inherent to human judgment, it offers substantial advantages in systematic and reproducible content analysis.

Analyses of video engagement parameters revealed significant positive correlations between VPI and DISCERN, JAMA, and GQS scores. This indicates that higher-quality videos tend to receive greater audience engagement. However, the absence of a significant association between the like ratio (%) and quality scores suggests that popularity does not necessarily align with informational accuracy or educational value. Similarly, studies on surgical education videos have reported considerable variability in the scientific accuracy of highly viewed content (2, 12, 13). Therefore, social media–based visibility indicators alone cannot be considered reliable proxies for content quality, emphasizing the need for rigorous academic and ethical review before such materials are integrated into educational curricula.

One of the major methodological strengths of this study is its cross-comparative design, which simultaneously evaluated both the mode of production (human vs. AI) and the mode of assessment (human vs. AI). This approach, distinct from previous studies in the literature, allowed for the analysis of the interaction between content source and evaluator type within a single research framework. Additionally, the use of three validated scales (DISCERN, JAMA, and GQS) enabled a comprehensive evaluation of educational quality, encompassing not only informational accuracy but also ethical transparency and user experience dimensions.

Nevertheless, this study has certain limitations. First, the sample consisted exclusively of English-language anesthesia education videos available on the YouTube platform, which limits the generalizability of the findings to other languages or platforms. Second, classification of videos as AI-generated relied on uploader disclosure; therefore, misclassification is possible, and the specific generative tools used were not consistently reported across videos. Third, only two human expert raters were included; although inter-rater reliability was high, larger and more diverse panels (including learners such as residents) may improve robustness and external validity. Fourth, ChatGPT-5 Plus scoring was based on transcripts and platform metadata rather than direct audiovisual review, and AI outputs may show minor variability across runs and with future model updates. Finally, as this study employed a cross-sectional design, the findings reflect model performance during the study period and may vary with subsequent algorithmic advancements.

## Conclusion

In conclusion, human-generated content demonstrated higher reliability and transparency than AI-generated videos, as reflected by significantly higher DISCERN and JAMA scores, whereas overall quality (GQS) did not differ significantly. When provided with standardized inputs consisting of transcripts and YouTube metadata, ChatGPT-5 Plus showed strong alignment with human expert ratings across all three instruments. These findings support the use of large language models as an adjunct tool for preliminary, transcript-based triage and quality screening, rather than a replacement for expert appraisal. Because transcript-based scoring does not capture audiovisual pedagogic features and minor output variability may occur, hybrid human–AI workflows and further validation with larger and more diverse rater panels are warranted.

## Data Availability

The raw data supporting the conclusions of this article will be made available by the authors, without undue reservation.
